# PI3K-C2α regulates Polycystin-2 ciliary entry to prevent kidney cyst formation

**DOI:** 10.1186/2046-2530-4-S1-O1

**Published:** 2015-07-13

**Authors:** JP Margaria, I Franco, A Ranghino, D Monteyne, M Chiaravalli, M Pema, C Campa, F Gulluni, D Perez-Morga, G Merlo, A Boletta, E Hirsch

**Affiliations:** 1 Department of Molecular Biotechnology and Health Science, University of Turin, Turin, Italy; 2 Department of Medical Sciences, Dialysis and Transplantation, Renal Transplantation Center "A. Vercellone", Turin, Italy; 3 Institut de Biologie et de Médecine Moléculaires (IBMM), Laboratoire de Parasitologie Moléculaire, Université Libre de Bruxelles, Charleroi, Belgium; 4 Division of Genetics and Cell Biology, Dibit San Raffaele Scientific Institute, Milan, Italy; 5 Center for Microscopy and Molecular Imaging-CMMI, Charleroi, Belgium

## Objective

PI3K-C2α is a regulator of vesicle recycling at the base of the primary cilium and is required for the targeting of ciliary components. Here we sought to understand whether PI3K-C2α is required for the targeting of Polycystin-2 to primary cilia and the consequent regulation of kidney cyst formation.

## Methods

Homozygous mutation of *Pik3c2a*, the gene encoding for PI3K-C2α, is embryonic lethal. Hence, the function of PI3K-C2α in kidney cilia has been studied both *in vitro*, in *Pik3c2a*-silenced IMCD3 cells, and *in vivo*, in *Pik3c2a*-heterozygous mice, using an Ischemia/Reperfusion model of renal injury.

## Results

PI3K-C2α resides at the recycling endosome compartment surrounding the primary cilium base where it controls the activation of Rab8, a key mediator of cargo protein targeting to the primary cilium. Consistently, partial reduction of PI3K-C2α is sufficient to impair elongation of the cilium both in *Pik3c2a*-silenced IMCD3 cells and in kidney tubules of *Pik3c2a*^+/- ^mice. Importantly, absence of PI3K-C2α impairs the Rab8-dependent transport of Polycystin-2 to cilia and produces an overactivation of proliferative pathways regulated by ciliary Polycystins, such as the mTOR and MAPK pathways. Both defects can be rescued by transfection of constitutively active Rab8. In line with defective Polycystin signaling, heterozygous deletion of PI3K-C2α in mice causes an overall deregulation of proliferative signals in response to Ischemia/Reperfusion-induced renal damage, and this condition predisposes to cyst development.

## Conclusion

These results indicate that PI3K-C2α is required for the transport of ciliary components like Polycystin-2 and that reduction in PI3K-C2α levels is sufficient to enhance susceptibility to cystic kidney disease.

